# Chip-Based Molecular Evaluation of a DNA Extraction Protocol for *Candida* Species from Positive Blood Cultures

**DOI:** 10.3390/microorganisms12010081

**Published:** 2023-12-31

**Authors:** Vittorio Ivagnes, Giulia Menchinelli, Flora Marzia Liotti, Elena De Carolis, Riccardo Torelli, Desy De Lorenzis, Cinzia Recine, Maurizio Sanguinetti, Tiziana D’Inzeo, Brunella Posteraro

**Affiliations:** 1 Dipartimento di Scienze Biotecnologiche di Base, Cliniche Intensivologiche e Perioperatorie, Università Cattolica del Sacro Cuore, 00168 Rome, Italy; vittorio.ivagnes@gmail.com (V.I.); desy.delorenzis@gmail.com (D.D.L.); cinzia.recine@gmail.com (C.R.); tiziana.dinzeo@unicatt.it (T.D.); brunella.posteraro@unicatt.it (B.P.); 2 Dipartimento di Scienze di Laboratorio e Infettivologiche, Fondazione Policlinico Universitario A. Gemelli IRCCS, 00168 Rome, Italy; giulia.menchinelli@policlinicogemelli.it (G.M.); floramarzia.liotti@policlinicogemelli.it (F.M.L.); elena.decarolis@policlinicogemelli.it (E.D.C.); riccardo.torelli@policlinicogemelli.it (R.T.); 3 Dipartimento di Scienze Mediche e Chirurgiche Addominali ed Endocrino Metaboliche, Fondazione Policlinico Universitario A. Gemelli IRCCS, 00168 Rome, Italy

**Keywords:** bloodstream infection, blood culture, *Candida* species, PCR assay, yeast blood chip, molecular detection

## Abstract

The diagnosis of *Candida* bloodstream infection (BSI) may rely on a PCR-based analysis of a positive blood culture (PBC) obtained from the patient at the time of BSI. In this study, a yeast DNA extraction protocol for use on PBCs was developed and evaluated with the molecular mouse (MM) yeast blood (YBL) chip-based PCR assay, which allowed us to detect nine medically relevant *Candida* species. We studied 125 simulated or clinical PBCs for *Candida* species. A positive correlation between the DNA concentration and colony-forming unit count was found for simulated (Spearman’s ρ = 0.58; *p* < 0.0001) and clinical (Spearman’s ρ = 0.23, *p* = 0.09) PBCs. The extracted DNA yielded positive results with the MM YBL chip assay that agreed with the *Candida* species-level identification results for 63 (100%) of 63 isolates from simulated PBCs and 66 (99.5%) of 67 isolates from clinical PBCs. The false-negative result was for one *C. tropicalis* isolate that grew together with *C. albicans* in PBC. None of the 30 (*Candida*)-negative clinical BCs included as negative controls yielded a positive result with the MM YBL chip assay. Our DNA extraction protocol for the *Candida* species couples efficiency and simplicity together. Nevertheless, further studies are needed before it can be adopted for use with the MM YBL chip assay.

## 1. Introduction

The *Candida* species remains an important cause of bloodstream infections (BSIs) in Europe and the United States of America (USA), with candidemia (defined as the presence of *Candida* cells in the patient’s blood) accounting for 1 in 10 BSIs from patients admitted to tertiary-care hospitals [1]. It is also probably the best-documented syndrome associated with invasive candidiasis [2]. Although *Candida albicans* is the most common agent, several non-*C. albicans Candida* (NCAC) species are causative agents of BSI worldwide [3], including (in alphabetic order) *C. auris* (which came to light in 2009; see [4]), *C. dubliniensis*, *C. glabrata* (*Nakaseomyces glabrata*), *C. guilliermondii* (*Meyerozyma guilliermondii*), *C. krusei* (*Pichia kudriavzevii*), *C. lusitaniae* (*Clavispora lusitaniae*), *C. parapsilosis*, and *C. tropicalis*, among others. Almost all these species can form biofilms [5,6], and this capability makes *Candida* BSI or other forms of invasive candidiasis very difficult to treat with conventional antifungal agents [7,8].

While blood culture (BC) represents the cornerstone of candidemia diagnosis, PCR-based assays, namely the BioFire FilmArray BCID2 panel (bioMérieux, Inc., Durham, NC, USA) and the ePlex BCID Fungal Pathogen panel (GenMark Diagnostics, Inc., Carlsbad, CA, USA), have been designed to identify various *Candida* species (7 and 11, respectively) taken directly from positive blood culture (PBC) samples [9]. In both assays, multiplexed nucleic acid extraction, amplification, and amplicon detection are performed automatically using all reagents contained in one pouch (FilmArray BCID2 panel) or cartridge (BCID-FP panel). The performance of these assays may, however, be suboptimal [9], perhaps due to the inefficient recovery of DNA from fungal (yeast) cells [10,11]. It should be recalled that the rigid cell walls of *Candida* (or other fungi) make sample preparation very complex, possibly requiring chemical, physical, or enzymatic steps before PCR amplification is performed [12].

In this study, we developed a DNA extraction protocol for clinical or simulated PBC samples for *Candida* species. The efficiency of this protocol was evaluated by testing the extracted DNA from each sample with a yeast blood (YBL) chip, which contained all reagents needed to perform real-time PCR in a molecular mouse (MM) instrument (Alifax S.r.l., Polverara, PD, Italy). Before this study, the YBL chip had obtained the CE-IVD (European conformity for in vitro diagnostic medical devices) mark for the detection of the above-mentioned *Candida* species in PBC samples.

## 2. Materials and Methods

### 2.1. Study Setting and Samples

This study was conducted at the clinical microbiology laboratory of Fondazione Policlinico Universitario A. Gemelli IRCCS, a large tertiary-care hospital in Rome, Italy. Part of this study has been presented at the 11th Trends in Medical Mycology (TIMM) held in Athens, Greece (20–23 October 2023).

A total of 155 BC samples, including positive and negative samples for *Candida* species, were studied. We simulated BCs (*n* = 63) as previously described [13,14], using clinical isolates (7 for each species) of *C. albicans*, *C. auris*, *C. dubliniensis*, *C. glabrata*, *C. guilliermondii*, *C. krusei*, *C. lusitaniae*, *C. parapsilosis*, and *C. tropicalis* that had been kept at −80 °C. Before use, each isolate was revitalized by culturing on Sabouraud dextrose agar (SDA) plates, and its identity was confirmed via MALDI-TOF mass spectrometry-based identification [15]. Each isolate’s suspension was prepared in phosphate-buffered saline (PBS), reaching a final inoculum of 50 to 100 cells/mL. Then, an injection volume consisting of 1.0 mL suspension and 9 mL human whole blood was used to fill a BacT/Alert BC bottle (bioMérieux, Marcy-l’Étoile, France). This allowed us to obtain a candidemia level of 5 to 10 cells/mL in each bottle. For clinical BC bottles (see below), simulated BC bottles were incubated in a BacT/Alert VIRTUO BC system (bioMérieux) instrument at 37 °C to allow microbial growth (i.e., until the bottles signaled positive). We also studied clinical BC bottles, namely BacT/Alert BC bottles obtained from hospitalized patients, that were positive for *Candida* species (*n* = 62), positive for bacterial species (*n* = 15), and negative for bacterial and yeast species (*n* = 15). All clinical BC bottles were obtained during the BSI diagnostic laboratory workflow that has been operating since 2016 [16], with microbial isolates that grew from PBC bottles identified via MALDI-TOF mass spectrometry as mentioned above.

Samples from both simulated and clinical BCs were serially diluted in PBS, and aliquots (100 μL) of each sample were plated onto SDA and/or *Candida* bromcresol green (BCG) agar to assess the colony-forming unit (CFU) count (expressed as number × 10^5^/mL) after incubating the plates at 37 °C for 48 h. Using BCG helped to differentiate the *Candida* species from each other in polymicrobial samples [17]. In parallel, each sample was subjected to an in-house DNA extraction protocol as described below or, for comparison purposes only, to a commercial DNA extraction protocol using one of the kits previously used by Alifax S.r.l. (e.g., Qiagen DNeasy Blood & Tissue kit) during the CE-IVD marking process (https://www.alifax.com/products/molecular-mouse/, accessed on 25 October 2023).

### 2.2. Development of a DNA Extraction Protocol for Candida Species

**Figure 1:**
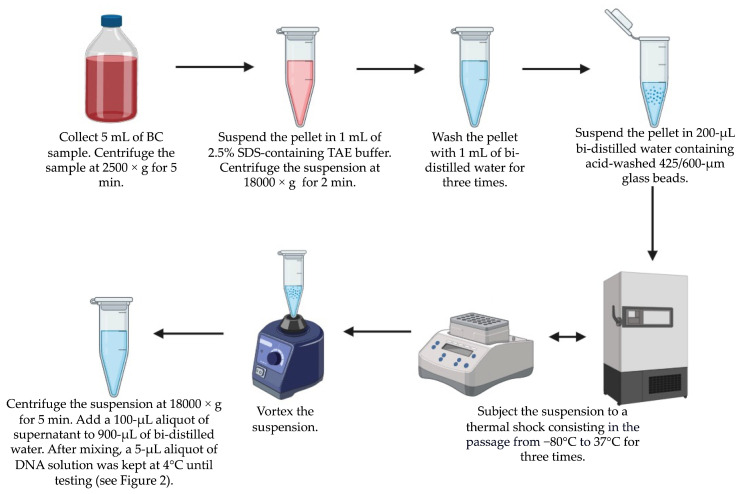
In-house protocol steps to extract DNA from PBC samples for *Candida* species. Once the process is complete, the DNA solution is available for testing within approximately 60 min of sample collection.

As detailed in Figure 1, a 5 mL sample from each PBC was processed according to a multistep protocol consisting of centrifugation, suspension in a TRIS-acetate-EDTA buffer, washes, mechanical lysis, thermal shock, vortexing, and final centrifugation to collect DNA-containing supernatant. The DNA solution (1 mL) in bi-distilled water was kept at 4 °C until testing with the MM YBL chip assay. Before use, a 1 μL aliquot of the DNA solution was used to determine the DNA concentration (expressed as ng/μL) with the NanoDrop ND-1000 spectrophotometer (Thermo Fisher Scientific, Rodano (Milan), Italy).

### 2.3. Testing Extracted DNA with the MM YBL Chip Assay

The MM YBL chip assay (MM YEAST BLOOD, ref SI 1701.0105, Alifax) used in this study relies on an on-chip real-time PCR technology developed previously [18,19]. It consists of a 6-well multiplexed reaction chip (i.e., YLB chip), which integrates temperature sensors and heaters, and a miniaturized instrument (i.e., MM), which thermally and optically drives the YBL chip during the PCR. The optical module includes independent optical channels for 6-carboxyfluorescein (6FAM), hexachorofluorescein (HEX), or carboxy-X-rhodamine (ROX) fluorescence reporters, which are excited through light sources and then monitored for fluorescence emission. The PCR reagents in the YBL chip include primers and fluorophore-labeled probes for the specific detection of *C. albicans*, *C. auris*, *C. dubliniensis*, *C. glabrata*, *C. guilliermondii*, *C. krusei*, *C. lusitaniae*, *C. parapsilosis*, or *C. tropicalis* DNA.

**Figure 2:**
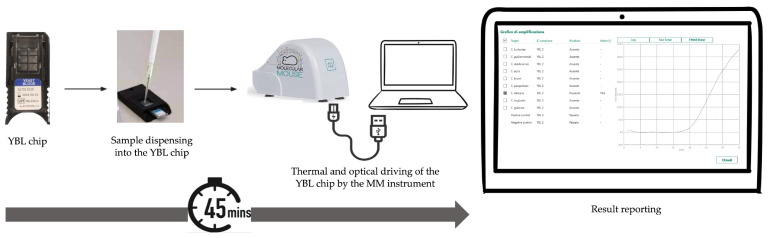
Workflow for testing a DNA sample with the MM YBL chip assay. It takes less than one hour from the time the sample-loaded chip is inserted into the MM instrument until the sample result is obtained.

As shown in Figure 2, a 5 μL aliquot of the DNA solution, obtained as described above, was loaded onto the YLB chip following the manufacturer’s instructions. At the end of the PCR, the MM instrument software captured and analyzed the fluorescence signal of each well within which the PCR reaction had occurred. The software produced a graphical output where positive results were expressed by cycle threshold (Ct) values, defined as the number of cycles at which the fluorescent signal exceeds the threshold for positive detections. The representative amplification curves for each *Candida* species targeted by the assay are depicted in Figure S1.

### 2.4. Data Analysis

The DNA concentration or CFU count values in PBC samples for *Candida* species, as well as PCR Ct values obtained via sample testing with the MM YBL chip assay, were expressed as mean ± standard deviation (SD). Positive percent agreement (PPA) and negative percent agreement (NPA), along with their respective confidence interval (CI), were calculated by comparing the MM YBL chip assay results with the culture-based results for the positive and negative BC samples for the *Candida* species, respectively. The differences in the PCR Ct values between the PBC sample groups were assessed using one-way analysis of variance (ANOVA) with Tukey’s multiple-comparison test. To investigate the relationship between DNA concentrations and CFU counts, Spearman’s correlation analysis was conducted for the BC samples, each positive for one *Candida* species (*n* = 120). All analyses were performed using the SPSS Statistics software version 24.0 (IBM Corp., Armonk, NY, USA) or the GraphPad Prism 9 software (GraphPad Software, San Diego, CA, USA), as appropriate. Statistical significance was set at a *p* of < 0.05.

## 3. Results

We studied 125 samples of simulated (*n* = 63) or clinical (*n* = 62) PBCs for medically important *Candida* species. The first group consisted of samples obtained through a BC simulation model with clinical isolates of *C. albicans* (*n* = 7) or NCAC species (*C. auris*, *C. dubliniensis*, *C. glabrata*, *C. guilliermondii*, *C. krusei*, *C. lusitaniae*, *C. parapsilosis*, and *C. tropicalis*; *n* = 7 for each species). The second group consisted of samples from BCs of patients with BSI caused by *C. albicans* (*n* = 20), *C. parapsilosis* (*n* = 17), *C. glabrata* (*n* = 12), *C. lusitaniae* (*n* = 4), *C. krusei* (*n* = 2), *C. tropicalis* (*n* = 2), *C. albicans* and *C. parapsilosis* (*n* = 2), *C. albicans* and *C. tropicalis* (*n* = 2), or *C. lusitaniae* and *C. parapsilosis* (*n* = 1). We also included samples from clinical BCs that were positive for bacterial species and negative for *Candida* species (*n* =15) and samples from clinical BCs that were negative for both bacterial and *Candida* species (*n* =15). All 30 samples served as negative controls.

Regarding simulated PBC samples, the number (×10^5^) of CFU per mL ranged from 90 to 165 for *C. albicans*, 21 to 168 for *C. auris*, 94 to 166 for *C. dubliniensis*, 87 to 173 for *C. glabrata*, 106 to 169 for *C. guilliermondii*, 51 to 112 for *C. krusei*, 87 to 184 for *C. lusitaniae*, 80 to 170 for *C. parapsilosis*, and 81 to 150 for *C. tropicalis*. Regarding clinical PBC samples, the number (×10^5^) of CFU per mL ranged from 0.8 to 138 for *C. albicans*, 17 to 153 for *C. glabrata*, 72 to 82 for *C. krusei*, 0.3 to 122 for *C. lusitaniae*, 0.3 to 149 for *C. parapsilosis*, and 0.07 to 167 for *C. tropicalis*. The lowest numbers of CFU per mL listed were for clinical PBC samples for more than one *Candida* species (*C. albicans* (0.8) and *C. tropicalis* (0.07), 1 sample; *C. lusitaniae* (0.3) and *C. parapsilosis* (0.3), 1 sample).

The DNA extraction protocol (Figure 1) was applied to simulated PBC samples, resulting in a DNA concentration (ng/µL) that ranged from 26.4 to 62.3 for *C. albicans*, 25.2 to 59.4 for *C. auris*, 19.9 to 64.1 for *C. dubliniensis*, 40.2 to 68.5 for *C. glabrata*, 32.8 to 61.3 for *C. guilliermondii*, 32.2 to 84.4 for *C. krusei*, 19.4 to 46.9 for *C. lusitaniae*, 20.8 to 98.5 for *C. parapsilosis*, and 21.9 to 67.4 for *C. tropicalis*. The same protocol was applied to clinical PBC samples, resulting in a DNA concentration (ng/µL) that ranged from 8.4 to 55.3 for *C. albicans*, 5.7 to 49.7 for *C. glabrata*, 19.5 to 22.4 for *C. krusei*, 6.5 to 24.8 for *C. lusitaniae*, 7.9 to 84.2 for *C. parapsilosis*, and 18.2 to 29.6 for *C. tropicalis*.

**Figure 3:**
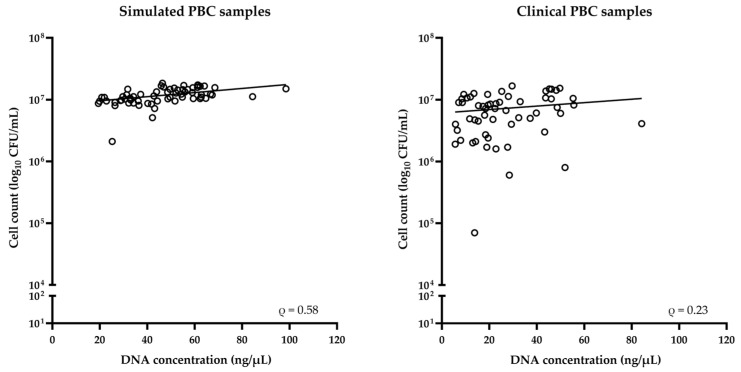
Relationship between DNA concentration and CFU count for simulated or clinical PBC samples for *Candida* species. According to Spearman’s correlation coefficient (ρ), a positive correlation was observed for both sample groups.

As shown in Figure 3, the DNA concentration values were assessed in relation to the CFU values from simulated (*n* = 63) or clinical (*n* = 57, after excluding five samples that grew two different species) PBC samples for *Candida* species. Thus, we found a positive correlation between the two groups of values for simulated (Spearman’s ρ = 0.58; *p* < 0.0001) or clinical (Spearman’s ρ = 0.23, *p* = 0.09) samples.

Table 1 summarizes the results obtained by testing the extracted DNAs from 155 BC samples with the MM YBL chip assay, as stratified by *Candida* species. For 63 (100%) of 63 isolates from the simulated PBC samples and 66 (98.5%) of 67 isolates from the clinical PBC samples (of which five grew two different species), the extracted DNA yielded a positive result for the *Candida* species to which each isolate belonged. The false-negative result was for one PBC sample for *C. tropicalis* (which also grew *C. albicans*). None of the 30 negative control samples yielded positive results with the MM YBL chip assay.

**Figure 4:**
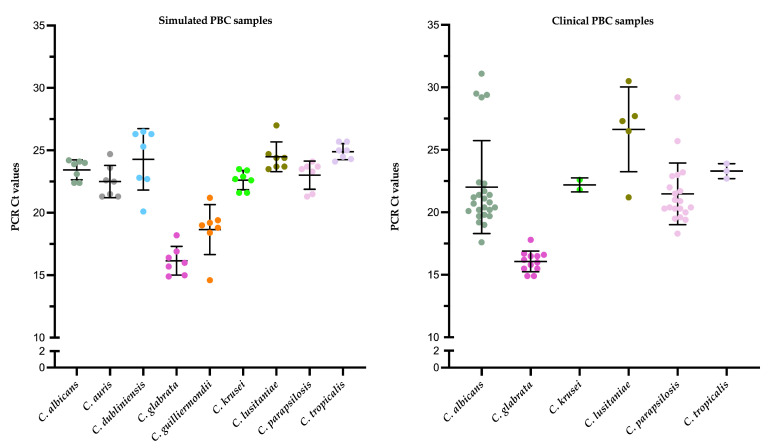
Distribution of PCR Ct values for simulated or clinical PBC samples with DNAs that were tested with the MM YBL chip assay. Values were grouped according to the *Candida* species detected. In each scatter dot plot (a different color is used to mark each *Candida* species), the central line indicates the mean Ct value, and the area between the top and bottom lines indicates the standard deviation value. There was statistical significance (*p* < 0.001) between groups of simulated or clinical PBC samples, as assessed using a one-way analysis of variance (ANOVA) with Tukey’s multiple-comparison test. Ct, cycle threshold.

Figure 4 shows the PCR Ct values for simulated or clinical PBC samples, respectively, according to the *Candida* species detected. In the first sample group, the mean (±SD) Ct value for all *Candida*-positive samples was 22.2 ± 3.0, and the mean (±SD) value for *C. albicans*-positive (23.4 ± 0.8), *C. auris*-positive (22.5 ± 1.4), *C. dubliniensis*-positive (24.3 ± 2.5), *C. glabrata*-positive (16.2 ± 1.1), *C. guilliermondii*-positive (18.7 ± 2.0), *C. krusei*-positive (22.6 ± 0.8), *C. lusitaniae*-positive (24.1 ± 0.5), *C. parapsilosis*-positive (23.0 ± 1.1), or *C. tropicalis*-positive (24.9 ± 0.6) samples differed significantly across each species (*p* < 0.001). In the second sample group, the mean (±SD) Ct value for all *Candida*-positive samples was 21.2 ± 3.9, and the mean (±SD) value for *C. albicans*-positive (22.0 ± 3.7), *C. glabrata*-positive (16.1 ± 0.8), *C. krusei*-positive (22.2 ± 0.6), *C. lusitaniae*-positive (26.6 ± 3.4), *C. parapsilosis*-positive (21.5 ± 2.5), or *C. tropicalis*-positive (23.3 ± 0.6) samples differed significantly from each other (*p* < 0.001).

Table 2 shows the results of the MM YBL chip assay for DNAs from PBC samples, respectively, extracted with the in-house protocol (described above) or with a commercial protocol (as provided by the Qiagen DNeasy Blood & Tissue kit). We found that the in-house protocol allowed us to obtain lower or equivalent PCR Ct values than those with the commercial protocol. The differences between the Ct values were only statistically significant with the simulated PBC samples for *C. dubliniensis* (*p* = 0.01) or *C. lusitaniae* (*p* = 0.00).

## 4. Discussion

We evaluated a DNA extraction protocol specifically developed for the *Candida* species from PBC samples via the MM YBL chip assay, which has recently been introduced into the landscape of PCR-based assays currently available for use in BSI diagnosis. Apart from one sample (false-negative result for *C. tropicalis*, one of two *Candida* species present in the sample), the results of the MM YBL chip assay were in full agreement with those of the identification of *Candida* isolates grown from the PBC samples studied. While the PCR Ct values differed significantly depending on the *Candida* species detected, thus reflecting the different number of yeast cells present in the samples before DNA extraction, the specificity of the MM YBL chip assay was such that no false-positive results occurred.

Despite the advent of detection systems that work directly on whole blood samples [20], PBCs have become an attractive biological matrix on which a PCR assay can be applied to provide positive (or negative) results for BSI pathogens targeted by an assay in a significantly reduced turnaround time [21]. It is unsurprising that the global market for in vitro diagnostics is increasingly expanding and that leading companies in the diagnostics sector, such as the Italian Alifax S.r.l. [22], have invested resources in developing systems to improve the laboratory diagnosis of infectious diseases. One system is MM, the world’s first portable real-time PCR platform that uses lab-on-chip cartridges (e.g., MM YBL chip) for the rapid (approximately one hour) analysis of microbial (bacterial or fungal) DNA targets (64 in total) starting from PBCs.

Unlike the BioFire FilmArray BCID2 and ePlex BCID Fungal Pathogen panels [9], where fungal DNA (for 6 and 11 detectable *Candida* species, respectively) is extracted and detected together in a single assay, the MM YBL chip assay works with fungal DNA (for 9 detectable *Candida* species) obtained from a separate (not included in the assay) extraction process, possibly using a commercial (e.g., Qiagen DNeasy Blood & Tissue kit; Zymo Research YeaStar Genomic DNA kit) protocol. Additional time is required before a DNA sample can be tested with the MM YBL chip assay. However, this apparent drawback is offset by the expectation of high-quality DNA (e.g., devoid of PCR inhibitors) [12], which is difficult to obtain using one-step (automated) protocols such as those integrated into the two widely adopted PCR panel assays [9]. We showed that the performance of the MM YBL chip assay was not compromised using DNA from the in-house (non-commercial) extraction protocol. The results (in terms of PCR Ct values) were in accordance with those obtained using the extraction protocol of the Qiagen DNeasy Blood & Tissue kit, which was used by the assay’s manufacturer (i.e., Alifax S.r.l.) at the time of CE-IVD marking. Starting from a PBC sample, the DNA extraction process with the in-house protocol took 60 min (much less than the 100 min with the Qiagen DNeasy Blood & Tissue kit). We also noted that the time required to complete the MM YBL chip assay using DNA extracted with the in-house protocol was shorter in most cases. Accordingly, the time from the PBC sample to the result (i.e., the time to the identification of a BSI-causing *Candida* species) may be reduced when the MM YBL chip assay is used in combination with the in-house DNA extraction protocol, rather than with a commercial DNA extraction protocol. This can be a non-negligible advantage, especially considering the high hands-on time required due to the several centrifugation steps in the proposed in-house protocol. Another potential advantage of using a non-commercial DNA extraction protocol like the one we developed is its lower cost than Qiagen DNeasy Blood & Tissue or other commercially available kits.

Over the past few decades, accumulated experience with manual (including in-house) or automated DNA extraction protocols for *Candida* (or other yeast) species has shown that the yield of DNA extracted from whole blood samples can be variable (limit of detection ranges from 10^0^ to 10^6^ CFU/mL for manual protocols and from 10^1^ to 10^6^ CFU/mL for automated protocols) [11]. This variability is imputable as to whether pretreatment (mechanical, enzymatic, chemical, physical, or thermal) or no treatment was applied to the sample before proceeding with DNA isolation from *Candida* cells [11]. Very recently, Menu et al. [10] used human blood samples artificially spiked with the *Candida* species (*C. albicans*, *C. glabrata*, *C. parapsilosis*, *C. tropicalis*, and *C. krusei*, for which inoculum sizes ranged from 0 to 10^8^ CFU/mL) to evaluate the efficiency of 11 automated DNA extraction protocols. After testing the extracted DNA from each sample with a real-time PCR assay using *Candida* species-specific probes, only one protocol proved to be the most efficient, especially for samples with the lowest inoculum sizes (10^1^ to 10^2^ CFU/mL) or spiked with *C. tropicalis* [10]. *C. tropicalis* is superior in forming biofilms than *C. albicans*, *C. parapsilosis*, or *C. krusei*, which may explain why the other 10 protocols were less efficient at extracting DNA from this *Candida* species [10].

The present study has strengths and weaknesses. We agree with Wickes and Romanelli [12] that caution is needed to prevent the DNA template from negatively influencing the sensitivity of PCR-based fungal diagnosis. Thus, acting according to the opinion of Bandehpour et al. [23], we optimized our experimental conditions to ensure adequate quality and quantity of DNA and to simplify the extraction process for *Candida* species. Others may not understand our choice to develop a DNA extraction protocol from PBC samples rather than whole blood samples from patients with candidemia. However, we believe the risk of false-negative detections in the clinical setting may be almost zero using PBCs. The quantity of CFU/mL in all but one (the sample that grew two *Candida* species) of the patient PBC samples in our study was such that the amount of extracted DNA yielded positive detections for the MM YBL chip assay, with Ct values much below 35 (i.e., the threshold above which a sample can be considered negative with the assay). Adopting an in-house *Candida* species DNA extraction protocol must deal with the fact that certification from various entities forces clinical microbiology laboratories to rely on standardized methods. In this context, it will be necessary to share our protocol with clinical microbiologists in Italy or other countries before it can be universally adopted for use with the MM YBL chip assay.

In conclusion, we developed a DNA extraction protocol for *Candida* species that couples simplicity and efficiency together and promises to be an alternative to commercial kit protocols currently used in combination with PCR assays for detecting BSI-causing *Candida* species. Future multicenter laboratory trials are expected to confirm the findings from the present study.

## Figures and Tables

**Table 1:** Characteristics of BC samples from which DNA was extracted and tested with the MM YBL chip assay.

Positive BC Samples for *Candida* Species (*n* = 125) ^1^			Testing Results for DNAs from *Candida*-Positive (*n* = 125) or *Candida*-Negative (*n* = 30) BC Samples ^2^			
Type of BC (No. of *Candida* Isolates)	DNA Concentration (Mean ± SD) Expressed as ng/μL	CFU Count (Mean ± SD) Expressed as Number × 10^5^/mL	TP/TP + FN	Positive Percent Agreement (95% CI)	TN/TN + FP	Negative Percent Agreement (95% CI)
*C. albicans*						
Clinical (24)	29.1 ± 13.5	67.0 ± 44.0	24/24 + 0	100.0 (86.9–100.0)	68/68 + 0	100.0 (94.7–100.0)
Simulated (7)	47.2 ± 14.1	121.9 ± 26.9	7/7 + 0	100.0 (64.6–100.0)	56/56 + 0	100.0 (93.6–100.0)
Combined (31)	32.7 ± 15.3	79.4 ± 46.6	31/31 + 0	100.0 (89.0–100.0)	124/124 + 0	100.0 (97.0–100.0)
*C. auris*						
Clinical (0)	NA	NA	NA	NA	NA	NA
Simulated (7)	44.6 ± 10.6	98.3 ± 47.9	7/7 + 0	100.0 (64.6–100.0)	56/56 + 0	100.0 (93.6–100.0)
Combined (7)	44.6 ± 10.6	98.3 ± 47.9	7/7 + 0	100.0 (64.6–100.0)	56/56 + 0	100.0 (93.6–100.0)
*C. dubliniensis*						
Clinical (0)	NA	NA	NA	NA	NA	NA
Simulated (7)	42.5 ± 18.3	122.7 ± 29.4	7/7 + 0	100.0 (64.6–100.0)	56/56 + 0	100.0 (93.6–100.0)
Combined (7)	42.5 ± 18.3	122.7 ± 29.4	7/7 + 0	100.0 (64.6–100.0)	56/56 + 0	100.0 (93.6–100.0)
*C. glabrata*						
Clinical (12)	25.9 ± 17.4	76.3 ± 41.0	12/12 + 0	100.0 (75.8–100.0)	80/80 + 0	100.0 (95.4–100.0)
Simulated (7)	54.2 ± 10.1	139.3 ± 29.7	7/7 + 0	100.0 (64.6–100.0)	56/56 + 0	100.0 (93.6–100.0)
Combined (19)	36.3 ± 20.4	99.5 ± 48.0	19/19 + 0	100.0 (83.2–100.0)	136/136 + 0	100.0 (97.3–100.0)
*C. guilliermondii*						
Clinical (0)	NA	NA	NA	NA	NA	NA
Simulated (7)	48.8 ± 10.4	133.1 ± 22.5	7/7 + 0	100.0 (64.6–100.0)	56/56 + 0	100.0 (93.6–100.0)
Combined (7)	48.8 ± 10.4	133.1 ± 22.5	7/7 + 0	100.0 (64.6–100.0)	56/56 + 0	100.0 (93.6–100.0)
*C. krusei*						
Clinical (2)	21.0 ± 2.1	77.0 ± 7.1	2/2 + 0	100.0 (34.2–100.0)	90/90 + 0	100.0 (95.9–100.0)
Simulated (7)	53.1 ± 18.1	93.6 ± 20.7	7/7 + 0	100.0 (64.6–100.0)	56/56 + 0	100.0 (93.6–100.0)
Combined (9)	46.0 ± 21.2	89.9 ± 19.5	9/9 + 0	100.0 (70.0–100.0)	146/146 + 0	100.0 (97.4–100.0)
*C. lusitaniae*						
Clinical (5)	13.3 ± 8.2	54.7 ± 49.8	5/5 + 0	100.0 (56.6–100.0)	87/87 + 0	100.0 (93.7–100.0)
Simulated (7)	32.6 ± 10.6	129.4 ± 35.7	7/7 + 0	100.0 (64.6–100.0)	56/56 + 0	100.0 (93.6–100.0)
Combined (12)	24.6 ± 13.6	98.3 ± 55.5	12/12 + 0	100.0 (75.8–100.0)	143/143 + 0	100.0 (97.4–100.0)
*C. parapsilosis*						
Clinical (20)	28.2 ± 18.1	56.3 ± 48.0	20/20 + 0	100.0 (83.9–100.0)	72/72 + 0	100.0 (94.9–100.0)
Simulated (7)	49.5 ± 25.3	113.1 ± 24.3	7/7 + 0	100.0 (64.6–100.0)	56/56 + 0	100.0 (93.6–100.0)
Combined (27)	33.7 ± 21.8	71.0 ± 49.6	27/27 + 0	100.0 (87.5–100.0)	128/128 + 0	100.0 (97.1–100.0)
*C. tropicalis*						
Clinical (3)	21.2 ± 5.6	50.5 ± 78.5	3/3 + 1	75.0 (30.0–95.4)	89/89 + 0	100.0 (95.9–100.0)
Simulated (7)	47.4 ± 20.7	114.9 ± 28.2	7/7 + 0	100.0 (64.6–100.0)	56/56 + 0	100.0 (93.6–100.0)
Combined (10)	37.9 ± 21.0	91.5 ± 58.1	10/10 + 1	90.9 (62.3–100.0)	145/145 + 0	100.0 (97.4–100.0)

TP, true positive; FN, false negative; TN, true negative; FP, false positive; NA, not available. ^1^ Include 62 samples from clinical PBCs. Overall, 5 of 62 samples each grew two *Candida* species (1 *C. albicans* and 1 *C. parapsilosis*, two samples; 1 *C. albicans* and 1 *C. tropicalis*, two samples; and 1 *C. lusitaniae* and 1 *C. parapsilosis*, one sample), thus resulting in 67 isolates of *Candida* species tested in total. Accordingly, DNA from each of the five samples was treated as if it had been extracted from two separate samples, each positive for one of the two *Candida* species in the sample. ^2^ For samples from clinical PBCs (*n* = 62), the number of TN results for each *Candida* species (e.g., *C. albicans*) targeted by the MM YBL-chip assay was calculated by adding 30 (i.e., the number of negative control samples) to a number (e.g., 38) that was obtained by subtracting the number of positive samples for that *Candida* species (e.g., 24) from 62 (i.e., the number of samples tested). For samples from simulated PBCs (*n* = 63), the number of TN results for each *Candida* species targeted by the YBL chip-based assay was calculated by subtracting 7 (i.e., the number of positive samples for that *Candida* species) from 63 (i.e., the number of samples tested).

**Table 2:** Comparison of MM YBL chip assay results obtained with *Candida* DNAs from in-house and commercial extraction protocols.

	PCR Ct Values (Expressed as Mean ± SD) ^1^ for DNAs Extracted from *Candida*-Positive BC Samples with the		
	In-House Protocol	Commercial Protocol	*p* Value
Simulated samples (no. of isolates) for			
*C. albicans* (7)	23.4 ± 0.8	24.7 ± 2.2	0.21
*C. auris* (7)	22.5 ± 1.4	22.0 ± 0.7	0.17
*C. dubliniensis* (7)	24.3 ± 2.5	25.2 ± 1.0	0.01
*C. glabrata* (7)	16.2 ± 1.1	19.6 ± 1.2	0.63
*C. guilliermondii* (7)	16.2 ± 1.1	19.2 ± 1.5	0.64
*C. krusei* (7)	22.6 ± 0.8	24.2 ± 1.1	0.36
*C. lusitaniae* (7)	24.1 ± 0.5	26.3 ± 3.2	0.00
*C. parapsilosis* (7)	23.0 ± 1.1	23.6 ± 0.8	0.22
*C. tropicalis* (7)	24.9 ± 0.6	25.9 ± 0.5	0.33
Total species (56)	22.2 ± 3.0	23.4 ± 2.9	0.60
Clinical samples (no. of isolates) for			
*C. albicans* (24)	22.0 ± 3.7	22.6 ± 3.4	0.72
*C. glabrata* (12)	16.1 ± 0.8	18.9 ± 0.8	0.85
*C. krusei* (2)	22.2 ± 0.6	23.7 ± 0.7	NA
*C. lusitaniae* (5)	26.6 ± 3.4	27.4 ± 1.2	0.22
*C. parapsilosis* (20)	21.5 ± 2.5	22.3 ± 2.1	0.57
*C. tropicalis* (3)	23.3 ± 0.6	25.1 ± 2.0	0.14
Total species (66)	21.2 ± 3.9	23.2 ± 2.6	0.30

Ct, cycle threshold; SD, standard deviation; BC, blood culture; NA, not applicable. ^1^ Values are stratified according to whether DNAs were extracted from simulated (*n =* 63) or clinical (*n =* 62) *Candida*-positive BC samples using the in-house or commercial protocol, respectively.

## Data Availability

Data may be available upon reasonable request.

## Supplementary Materials

The following supporting information can be downloaded at: https://www.mdpi.com/article/10.3390/microorganisms12010081/s1, Figure S1: Real-time PCR results obtained with the MM YBL-chip assay, showing representative amplification curves for each *Candida* species targeted by the assay.
