# Supplementary material for: Chip-Based Molecular Evaluation of a DNA Extraction Protocol for Candida Species from Positive Blood Cultures
Source: Microorganisms. 2023 Dec 31;12(1):81. doi: 10.3390/microorganisms12010081 (PMC10821462; doi:10.3390/microorganisms12010081)
Supplement: Supplementary file 1 [file microorganisms-12-00081-s001.zip › microorganisms-2717685-supplementary.pdf]

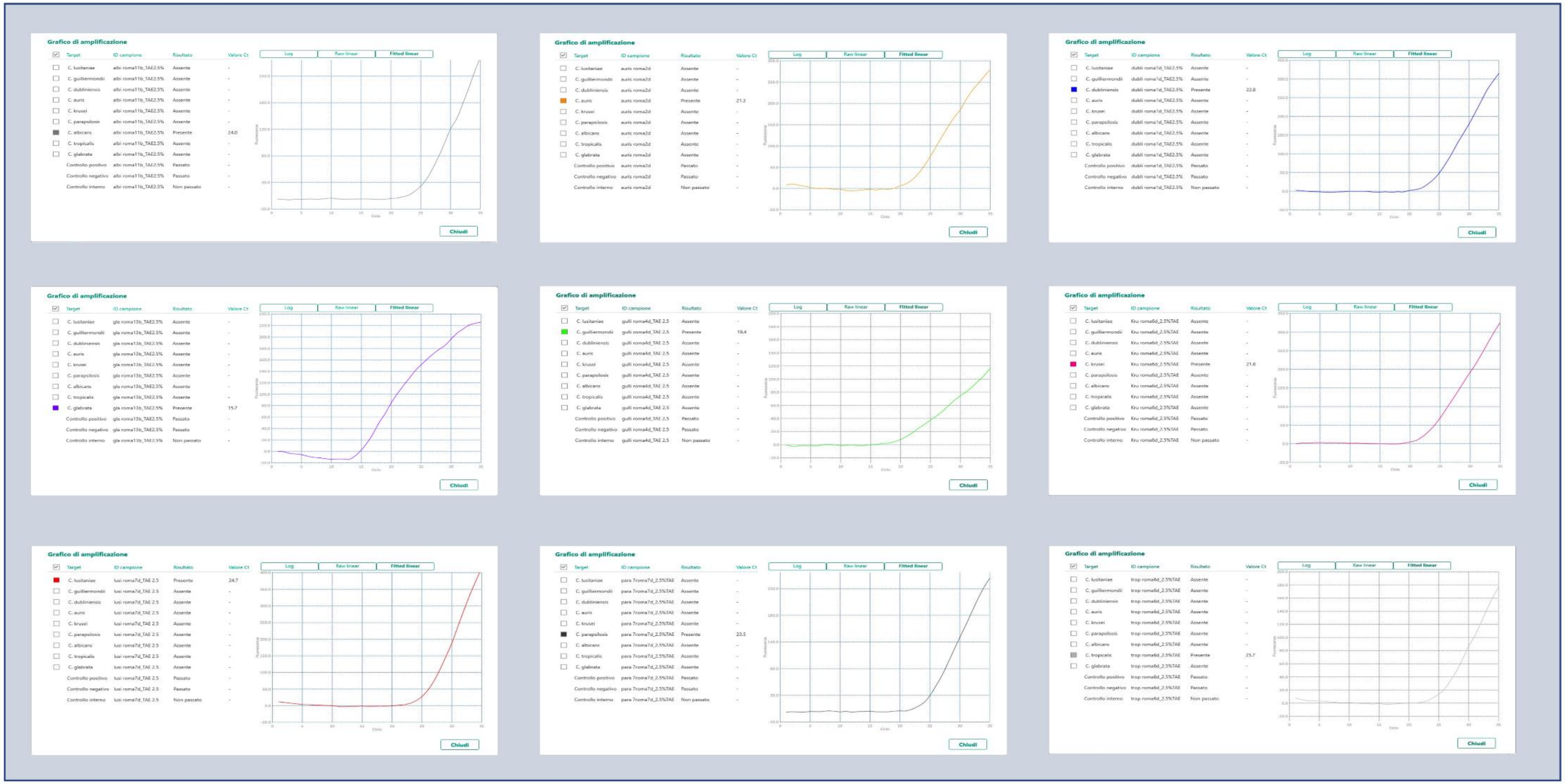

Figure S1. Real-time PCR results obtained with the MM YBL-chip assay, showing representative amplification curves for each *Candida* species targeted by the assay. A cycle threshold value (i.e., number of cycles at which the fluorescence signal crosses the threshold for positive detections) is shown from each fluorescence-*versus*-time sigmoidal curve.
